# DIC image reconstruction using an energy minimization framework to visualize optical path length distribution

**DOI:** 10.1038/srep30420

**Published:** 2016-07-25

**Authors:** Krisztian Koos, József Molnár, Lóránd Kelemen, Gábor Tamás, Peter Horvath

**Affiliations:** 1 Synthetic and Systems Biology Unit, Hungarian Academy of Sciences, BRC, Szeged, Hungary; 2 Institute of Biophysics, Hungarian Academy of Sciences, BRC, Szeged, Hungary; 3 MTA-SZTE Research Group for Cortical Microcircuits, Department of Physiology, Anatomy and Neuroscience, University of Szeged, Szeged, Hungary; 4 Institute for Molecular Medicine Finland (FIMM), University of Helsinki, Helsinki, Finland

## Abstract

Label-free microscopy techniques have numerous advantages such as low phototoxicity, simple setup and no need for fluorophores or other contrast materials. Despite their advantages, most label-free techniques cannot visualize specific cellular compartments or the location of proteins and the image formation limits quantitative evaluation. Differential interference contrast (DIC) is a qualitative microscopy technique that shows the optical path length differences within a specimen. We propose a variational framework for DIC image reconstruction. The proposed method largely outperforms state-of-the-art methods on synthetic, artificial and real tests and turns DIC microscopy into an automated high-content imaging tool. Image sets and the source code of the examined algorithms are made publicly available.

The simplest of all optical microscopy techniques is the brightfield microscopy that was invented by Anton van Leeuwenhoek^1^ in the 17^th^ century as an improved version of the modern microscope developed by Robert Hooke^2^. Phase contrast microscopy was proposed in the early 1930s by Fritz Zernike who was awarded the 1953 Nobel Prize in Physics for his invention^3^. Phase contrast microscopy is interference-based and allows the study of the internal structure of living cells. Two years later, in 1955, Georges Nomarski established the theoretical basis for differential interference contrast (DIC) microscopy^4^ that gains information about the optical path length of the sample and shows features that are invisible in a brightfield microscope. However, label-free techniques are unable to selectively visualize subcellular components, processes, or localization of proteins which generated a demand for new methods in microscopy.

In 1994 the successful expression of green fluorescent protein in living organisms brought fluorescence to the forefront^5^. Proteins can be stained (labeled) by fluorescent substances which show their localization inside the cells. Fluorescent images are quantitative, thus can easily be analyzed by suitable software. Despite their advantages, phase contrast and DIC are qualitative methods. Although these images are easy to understand for the human eye, conventional algorithms are not applicable when it comes to image processing. However, label-free techniques have advantages over fluorescent microscopy: namely, cells can be observed without staining, so these techniques are not phototoxic and there is no need for chemical fixation of the sample in contrast to numerous fluorescent staining protocols. Thus, label-free techniques allow live cell analysis in a physiologically more relevant way.

Nowadays, advanced label-free techniques are also used for examination of biological samples. Quantitative Phase-contrast Microscopy^6^,^7^,^8^ and optical diffraction tomography^9^,^10^ also gain information about the phase of cells. Raman microscopy^11^,^12^ can reveal the location and amount of different constituents. Our focus here is DIC microscopy due to its cost-effectiveness, widespread use in laboratories, and possibility to perform depth-resolved imaging similar to confocal fluorescence microscopy.

DIC microscopy images can be converted to show the optical path length distribution using the image formation model. Image formation in a DIC microscope starts as a light ray enters into a polarizer that creates plane polarized light. A Wollaston prism splits the light into two perpendicularly polarized light rays which are focused on the specimen by the condenser lens. These two light rays pass the sample by a minute shear. The distance between the rays, i.e. the shear distance, is smaller than the spatial resolution of the microscope’s objective. Materials with different refractive indices and the specimen’s thickness generate a phase shift between the light rays. Next a second lens, i.e. the objective transmits the lights to another Wollaston prism that recombines them. Finally, the second polarizer, called the analyzer creates plane polarized light perpendicular to the light of the first polarizer. The two light rays interfere at this point and generate a contrast image^4^. Usually the microscope is set to a positive bias by shifting the first DIC prism. In case of positive bias, the background in a DIC image is mid-gray and the optical path length differences are bright on the one side and dark on the other side of the objects. However, the microscope can be set to extinction based on the position of the first prism, when regions having large refractive indices and thickness gradients appear as bright highlights on a black background. Shifting the first prism to the opposite direction generates negative bias where intensities of the gradient features are reversed.

The prisms are often called DIC or Nomarski prisms. The Nomarski prism can be considered as the combination of the condenser or the objective lens with a Wollaston prism. The interference plane of a Nomarski prism is placed a few millimeters outside of its center, thus placing the specimen in the interference plane makes the use of lenses unnecessary.

The mathematical description of the DIC image formation model was described in several recent studies^13^,^14^,^15^. In this paper we use a linear approximation which considers the image formation as the convolution of the original object information (optical path length distribution) *I* by the Point Spread Function (PSF) of the DIC microscope. In linear models the PSF is often chosen to be the first derivative of a Gaussian function or the difference of two Dirac delta functions^16^,^17^. Images are considered as real valued intensity functions parameterized by image coordinates (*x*,*y*) on the domain Ω. The convolution is written as a local integral which has a window *W* = [−*d*,*d*] × [−*d*,*d*], where 2*d* + 1 is the window size and (*ξ*,*η*)∈*W*. Furthermore, let *d*Ω = *dxdy* and *dW* = *dξdη* represent the infinitesimal area elements of the integrals in the image and in the local domains, respectively. The shear direction is denoted by a unit vector ***u*** = [*u v*]^*T*^,(*u*^2^ + *v*^2^ = 1). The model is generalized with an appropriately chosen kernel function *K* and becomes:


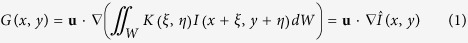


Note that the integral in equation (1) is parametric w.r.t. the image coordinates *x* and *y*.

The presented linear image formation limits the accuracy by ignoring diffraction and partial coherence effects. However, in Supplementary Discussion 1 we compare simulated DIC images generated by both the linear model and *microlith*^18^, an optically accurate simulation tool. Our comparisons show that the differences are visually negligible and the correlations between images of these two kinds are very high, typically between 0.96–0.99.

Here we propose a novel DIC reconstruction algorithm and give the most comprehensive overview of existing methods along with a broad range of comparisons. Furthermore, the tools used for DIC image reconstruction are made publicly available. Our new framework is based on energy minimization. The energy function uses a term that forces similarity to the original DIC image and a total variation-based regularization term. The first utilizes the PSF of the DIC microscope. The PSF is incorporated into our model by local integrals. We show that the derivation operation can be moved from the kernel to the image, which significantly accelerates the computations. Several other reconstruction methods are also described. The algorithms’ source codes are provided with the paper. We compare the reconstruction quality using three different test sets that are made publicly available. The algorithms open up the possibility of using DIC as a quantitative imaging modality.

## Results

In this section we describe our variational framework^19^,^20^ for DIC image reconstruction. The quality of the algorithm is tested on three different datasets that we made publicly available in the Broad Bioimage Benchmark Collection (BBBC)^21^. The first data set consists of synthetic images and a generated PSF based on the image formation model. The second set consists of real DIC microscopy images of micro-structures with known dimensions. The structures are fluorescent which can be used as a ground truth. Finally, the third data set consists of Chinese Hamster Ovary (CHO) cells with hand-segmentation ground truth. The results are compared to 6 other DIC reconstruction algorithms. The algorithms include three Fourier transform based ones, namely the Hilbert transform^17^,^22^, the Wiener filtering^17^,^23^ and an inverse filtering technique^24^ (referred to as Yin), two linear programming approaches, which are the sparseness-enhanced multiplicative update^16^ (SEMU) and a second order cone program^16^ (SOCP), and an earlier variational framework^25^ (referred to as Feineigle). The algorithms are described in detail in the Methods section. The MATLAB (Mathworks, Natick, MA, USA) source code of all the discussed algorithms and the result images of the proposed variational framework are publicly available on www.highcontentanalysis.org.

After reconstructing the DIC images by each algorithm, we compute the similarity of the results to the corresponding ground truth images. After removing the meaningless negative values, we normalize both the result and the ground truth images between 0 and 1 to compare reconstruction quality. A general approach is to calculate the Mean Squared Error (MSE) between the result and the ground truth images. A lower MSE value means better reconstruction. Higher pixel intensity differences increase the MSE value more than smaller differences. We remark that other conventional metrics such as correlation, norm and Mean Average Error gave the same ranking of the algorithms.

In real cases when no ground truth is available, comparing the convolved result image to the input DIC image might be the only way to measure quality. However, it can be a misleading metric because of the imperfect PSF, the noise and the optional smoothness term. In the real tests we show two problem-specific ways of obtaining ground truth images for real DIC images.

### Variational framework

The goal of DIC reconstruction algorithms is to reconstruct *I* given *G* and *K*. In a variational framework an energy function has to be defined. The energy function usually has the form of:


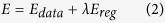


We propose a model, where *E*_*data*_ term has low value when *I* convolved with *K* is similar to *G*. *E*_*reg*_ ensures spatial regularization to have locally smooth regions where *G* is smooth (i.e. there are no edges on the original image). To find the minimizer, the variable *I* is usually updated in the direction of the gradient of the function.

We propose the following variational framework. The energy function is formulated as^26^:


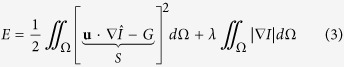


The data term ensures that the energy is low when the reconstructed image convolved by the PSF is similar to the input DIC image. Our choice of the smoothness term is the Total Variation (TV) term, where *λ* is a weight function, providing trade-off between data-driven reconstruction and denoising. TV denoising model^27^ preserves discontinuities, i.e. its associated gradient descent equation does not smooth across edges. The energy function is minimized by the gradient descent method. The gradient descent iteratively takes steps proportional to the negative gradient. To define the steps we calculate the Euler-Lagrangian (EL) equations. The complete derivation of the EL equations can be found in Supplementary Discussion 2, thus we confine to presenting only the main steps of the calculations here.

The EL equation of the data term contains the local derivatives of the perturbation function under local integrals. The analytical solution of the EL equations is complex. To overcome the difficulty, we use Taylor series. Let ∂_*x*_ and ∂_*y*_ denote the partial derivatives. Assuming that lower order terms contribute significantly more than higher order terms, for sufficiently small local integrals the ‘bilinear’ approximation of the EL equation can be formed as:


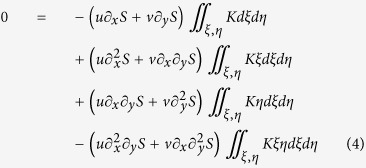


The EL equation of the TV smoothness term is defined pointwise as follows:


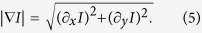


The finite kernel function *K* can be arbitrarily chosen and used in the equations at this point. Simplifications can be made by using special kernels. The first choice is a rotationally symmetric kernel *K*_*R*_. In case of *K*_*R*_, the one dimensional slices of the function along the parameter lines *x* or *y* become even functions, hence all terms having odd *m* or *n* in the weight function are eliminated and the bilinear approximation in equation (4) is reduced to ‘piecewise constant’ approximation:


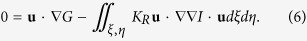


Equation (6) is given after multiplication by the weight factor 
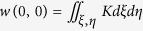
. ∇*G* is the gradient of the DIC image, ∇∇*I* is the Hessian of the primary image. Note that rotational symmetry is not a necessary condition, axis symmetry with axis aligned to the shear direction and going through the origin of the local integration window is sufficient.

However, the DIC PSF and its approximations do not satisfy the requirements. By moving the calculations of the derivations from the image to the kernel function with the parameter transformations *ξ* → *ξ* − *x*, *η* → *η* − *y* to the local integral in equation (1), its equivalent becomes:


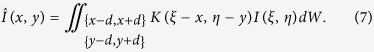


This form allows the use of the correct PSF. The partial derivatives become more complex, but higher order terms can be cancelled by using a kernel function *K*_0_ that has all derivatives zero on the boundary. Note that infinite and bounded kernels automatically satisfy this property. The rationale behind using equation (6) (i.e. leaving derivations on the image and using the integral of the PSF as kernel) is that it requires less calculation. Although the partial kernel derivatives can be precomputed, using all three of them for local integrations is computationally more expensive than calculating image derivatives. Hereafter this algorithm is referred to as the proposed one.

### Synthetic dataset

For the quality test of the algorithms on the synthetic dataset we have created 20 ground truth images of simple shapes, e.g. ellipse, rectangle or pentagon, and their rotated versions. The images are grayscale, but 15 of them contain only the minimum and maximum intensities, i.e. they can be considered as binary. The other 5 contain a range of different intensities. The simulated DIC images are created by convolving the ground truth images with an estimated PSF which is included in the dataset. The PSF we used is the difference of two Dirac delta functions which is a close approximation of the first derivative of the Gaussian function with *σ* = 0.5. The generated DIC images and the PSF are the inputs of the algorithms. The mentioned *σ* value assumes ideal conditions which are not present in a microscope. However, since some of the algorithms do not support any other PSF, *σ* = 0.5 allows us to perform a fair comparison. Furthermore, we have a noisy test set by adding 20 dB noise to the images.

**Figure 1:**
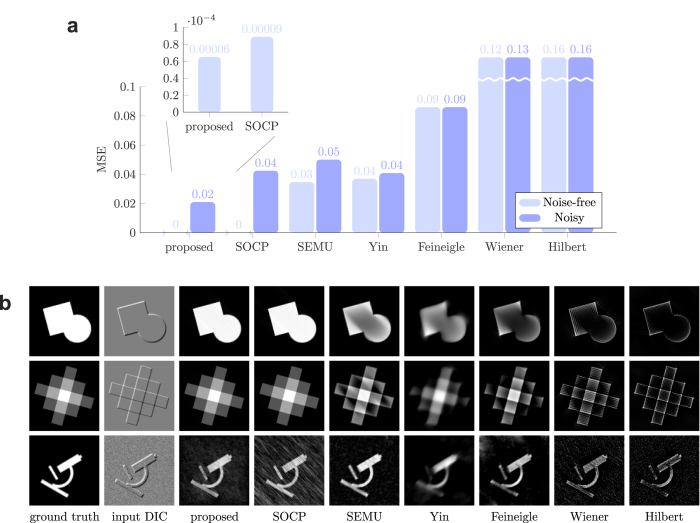
Comparison of the algorithms on synthetic data. **(a)** The bars show the average Mean Squared Error values. The algorithms are ordered based on their performance on the noise-free data. The enlarged part for the first two algorithms has different scale. **(b)** Example synthetic ground truth images, simulated DIC images and reconstructions.

Figure 1a shows the average MSE values of the algorithms in both noise-free and noisy cases. Different images are weighted equally regardless of the number of rotations. Figure 1b shows example images. In the noise-free images both the proposed and the SOCP algorithms seem to provide a desired result. Other algorithms are usually unable to reconstruct bright regions in the middle of larger objects, thus may only reconstruct the edges. The noisy tests change the quality ranking based on the MSE values, and the Yin algorithm performs remarkably well compared to SOCP. Overall, the best few algorithms, namely the proposed, the SOCP, the SEMU and the Yin algorithms perform well in general, but the quality of the result depends on the object and the noise level.

### Fluorescent artificial structures

For the quality test of the algorithms on micro-structures we created polymerized fluorescent surface-attached objects. Our dataset of polymerized objects consists of 6 different shapes: squares, square-shaped blocks of increasing height (referred to as step-like structures), triangles, rings, circles and hangar-like forms. The surface-attached test structures were polymerized by two-photon polymerization (TPP)^28^ on a custom-developed optical system constructed around a Zeiss Axiovert 200 inverted microscope^29^. In this process the beam of an ultrafast laser (C-Fiber A 780, Menlo Systems GmbH, Germany, Δ*τ* = 100 fs, *λ* = 785 nm, 100 MHz repetition rate) was focused into an ~18 μm thick layer of SU-8 2007 photoresist (Microchem, Newton, MA, USA) mixed with rhodamine 6G by a 100x oil immersion objective (NA = 1.25, Zeiss Apochromat). The sample was mounted on and translated by a piezo translation system (model P-731.8L in X−Y and P-721.10 in Z, Physik Instrumente, Germany) in 3 dimensions during the laser illumination. The photoresist layer was produced by spin coating 30 μL of SU-8 on a 170 μm thick cover slip and soft-baking it at 95 °C for 10 minutes to remove its solvent. For the step-like structures only one laser beam was used, but for the other structures the original single laser beam was split into four beams with a spatial light modulator (SLM, Pluto NIR, Holoeye GmbH, Germany) in order to produce four identical structures in a rectangular arrangement^30^. The power of each beam was set to 6 mW, and the scanning speed was set to 80 μm/s for all structures. After illumination the sample was baked again for 10 minutes at 95 °C to complete the polymerization, then developed in its matching developer (mr-Dev 600, Microchem, Newton, MA, USA) to remove the non-polymerized surplus and finally rinsed with ethanol and dried in a stream of nitrogen. The sample was immersed in oil with a refractive index of 1.515 (type DF, Cargille, Cedar Grove, NJ, USA), because the phase shift at the edges would be bigger than what DIC could correctly visualize. We have designed squares and step-like structures with a side length of 20 μm, rings with an outer and inner diameter of 20 μm and 13 μm, respectively, circles with a diameter of 20 μm, triangles with a side length of 20 μm and hangar-like structures with a tilt angle of about 9.5 degree and a width of 20 μm. We took both fluorescent and DIC images of the objects by an Olympus Cell-R microscope which can work in both modes without having to move the stage, thus the objects remain in place for the two modalities. Fluorescent microscopy can be considered quantitative and for thin samples the recorded intensity is linearly proportional to the thickness of a homogenous sample. We compare the reconstructions of the qualitative DIC images to the fluorescent ones. We calculated the MSE, but any other metrics described in Results section may be used. The total number of image pairs taken of the artificial samples was 60.

**Figure 2:**
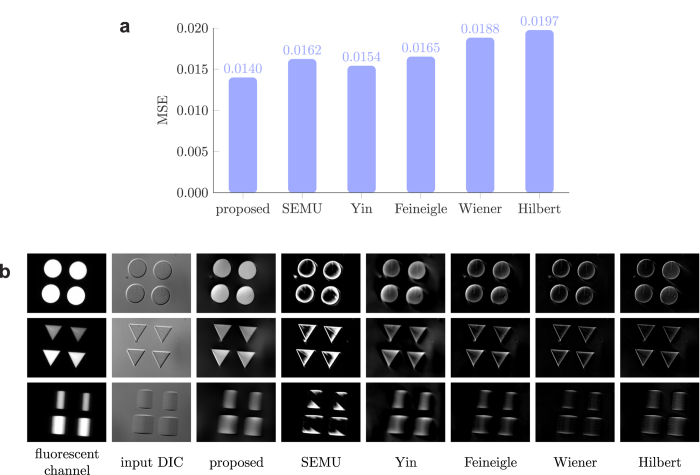
Reconstruction results of the algorithms on polymerized structures. **(a)** Average Mean Squared Error values between the fluorescent and the reconstructed images of artificial micro-structures. **(b)** Example images of micro-structures in fluorescent channel and in DIC mode and their corresponding reconstructions.

Figure 2a shows the average MSE values for the different reconstruction algorithms run on the artificial structures dataset. The SOCP algorithm is not included due to its excessive runtime. Quality ranking of the algorithms is very similar to that described in the synthetic dataset. Figure 2b shows some DIC and the corresponding fluorescent images with the results of the different algorithms. Again, it is obvious that some of the algorithms reconstruct only the edges of the objects. It is an issue particularly for the hangar-like objects (bottom row), where the shape is designed to test whether small changes are visible in the image.

### Cell images

The third quality test of the algorithms consists of 60 DIC images of CHO cells. The images were taken at the time when the cells had just started their attachment to the bottom of the dish. We made hand-segmentation of all the images which we use as ground truth for quality measurement of the reconstructions. Since the reconstructions are not binary images, comparing them to the binary hand-segmentations is not a reliable quality measurement method. Segmentation is a different and broad area of image processing and is out of the focus of the current work. Basic segmentation algorithms find a global threshold value derived from the examined image’s histogram. To overcome the problem of thresholding Receiver Operating Characteristics (ROC) Area Under Curve (AUC) is used. To calculate the AUC, the result image was thresholded by every possible value and both the True Positive Rate (TPR) and the False Positive Rate (FPR) were determined for every threshold value. The ROC space is then defined by FPR and TPR as *x* and *y* axes, respectively. The AUC is the area under the FPR/TPR plot. Higher AUC value indicates better reconstruction. An AUC value of 1 indicates that the result is equivalent to the ground truth and the thresholded image is always the expected regardless of the threshold value used.

**Figure 3:**
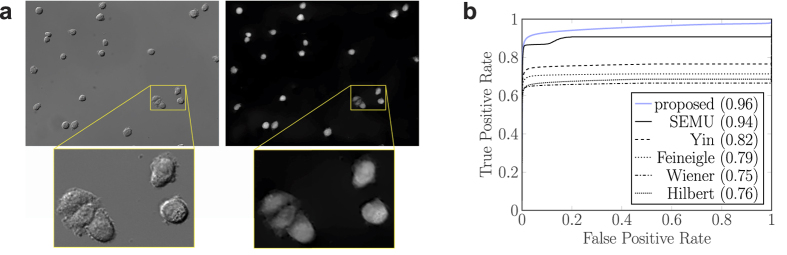
Comparison of algorithms on cell images. **(a)** Example DIC image of CHO cells and its reconstruction with the proposed algorithm. Yellow boxes show enlarged parts of the images. (**b**) Average ROC Curves of cell images. The numbers in the legend show the AUC values.

Figure 3a shows a result image of the proposed algorithm and the corresponding DIC image and hand-segmentation. All of the algorithms tested are able to reconstruct DIC images that can be subject to further processing. Some images are blurred or artifacts, bright lines may appear at the edges of the cells in the shear direction. Figure 3b shows the average ROC curves for every algorithm. The SOCP algorithm is not included due to its excessive runtime. The proposed variational framework has the highest AUC value, followed by SEMU and their differences to the rest of the algorithms are remarkable.

**Figure 4:**
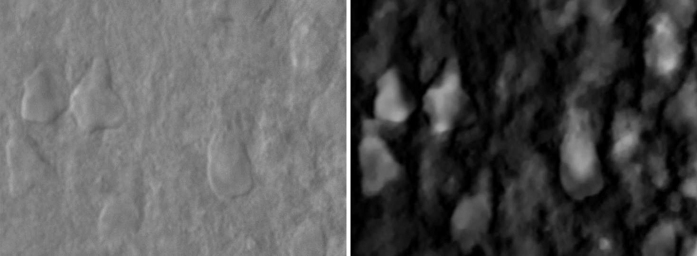
Example brain tissue image and its reconstruction with the proposed algorithm.

It is important to note that the proposed algorithm is tested to reconstruct DIC images of tissue samples. A DIC image of a brain tissue and the corresponding reconstruction is shown in Fig. 4. Although extracellular matrix, axons and dendrites can be seen as well in DIC images, the cell bodies are clearly visible in the reconstructions as bright intensity regions. Extending the algorithm from slices to volumes is a future work.

### Interpretation of the reconstructions

**Figure 5:**
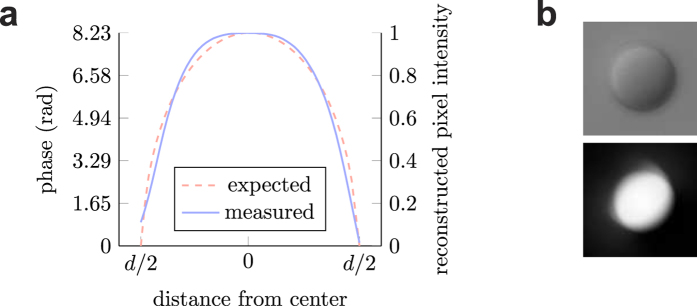
Calibration on microbeads. **(a)** Line plot of both the expected bead phase and the reconstructed beads, averaged from four directions, where d is the diameter of the bead. **(b)** An example DIC image of a microbead and its reconstruction.

The reconstructed images of the algorithms show the lateral morphology of the examined samples. Furthermore, the reconstructed images show proportions of the optical path lengths relative to the surrounding medium. The lowest value in the image means that the corresponding light rays went through no other material but the surrounding medium. The highest value represents the longest optical path length through the sample. To obtain the exact optical path length distribution, the microscope system has to be calibrated and a quantitative measurement can be performed as follows: The illumination has to be set to a constant value. The microscope software should be set that it does not allow histogram operations (e.g. stretching) on the image. Then an object with known *Z* dimension should be imaged where the refractive indices of both the object and the surrounding medium is known. Finally, assuming the discussed linear image formation model, a unit change in pixel intensity in the reconstructed image can be expressed in terms of optical path length change, which then can be used to measure further samples. However, the noise and diffraction effects cannot be accounted from a single image and can introduce errors. In Fig. 5 we show the result of the calibration on polystyrene microbeads (with a refractive index of 1.595). The diameter of the microbeads used is 9 μm (Polybead, Polysciences, Warrington, PA, USA). The beads are immersed in oil with a refractive index of 1.515. The DIC images of 16 beads were reconstructed with the proposed algorithm. Then the line profiles from four directions, horizontal, vertical and two diagonals of the reconstructed bead images were averaged, scaled and compared to the theoretical phase profile. The line plot of the measured data in Fig. 5a shows flat-top behavior due to the slight smoothing effect of the TV term (with *λ* = 0.1), that high jumps are preserved and small differences (in this case, the middle region of the reconstructed bead) are flattened. The blurring effect in the bottom of Fig. 5b is caused by diffraction due to the sphericity, a nonlinear effect that is not reconstructed correctly by linear models. However, the correlation of the measured and expected data points is 0.9893. The scaling factor can be used to convert the reconstruction of other objects in the medium to phase.

## Discussion

In this paper we describe 7 different DIC reconstruction algorithms of 3 types: inverse filtering algorithms, linear equation solvers and energy minimization frameworks. The source code of the algorithms is made publicly available on www.highcontentanalysis.org. The algorithms are tested on 3 image sets, namely on a synthetic dataset with a generated PSF, on real DIC images of fluorescent polymerized micro-structures and on images of cells in biological samples with hand-segmentation. The image sets are made publicly available in BBBC. The reconstructions are compared to the ground truth images of the corresponding sets. Every algorithm is able to reconstruct DIC images so that the result image is visually acceptable. Some techniques generate artifacts like bright stripes in the shear direction. When it comes to processing thousands of images, small errors can accumulate resulting in false segmentation. This paper along with the publicly available source code provides a basis for automated high-throughput label-free microscopy. The provided implementations can be used to convert the qualitative DIC images into quantitative ones after calibrating the system. The converted, quantitative DIC images are similar to what other quantitative phase imaging techniques provide with the advantage that DIC is widely available and a cost-effective solution. The reconstructed images are appropriate for morphological analysis or tracking of living cells. Furthermore, a fluorescent channel is saved which can be used to stain specific compartments or speed up the acquisition.

## Methods

### Hilbert transform

The Hilbert transform^17^,^22^ is considered to be the first algorithm used to reconstruct DIC images. The 1D Hilbert transform *H* of a function *f* is the convolution of the function by 1/*πx*:


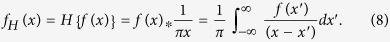


The required convolution in Fourier space is a multiplication. In 2D the spatial frequencies *k* and *l* that define the signum function have to be perpendicular to the DIC shear direction. Let *H* now denote the 2D Hilbert transform and **F** denote the Fourier transform. The real part of the inverse Fourier transform gives the resulting Hilbert transformed image:


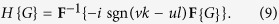


### Wiener filtering

If we consider the image formation model written in equation (1) as a convolution with the DIC PSF, the determination of *I* would be a simple division in Fourier space of the DIC image with the PSF. However, dividing by zero would produce large errors. Wiener filtering^17^,^23^ regularizes the equation by a small term based on the signal S and noise N ratio:


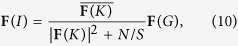


where the overline is the conjugate transpose.

### Inverse filtering

Wiener filtering is one technique to avoid division by zero in Fourier space. If we add regularization terms to the objective function, they can also prevent that values close to zero occur in the divisor. In such an approach^24^ a smoothness and a sparsity term is present in the objective function and the equation is solved in frequency space. In the original paper^24^ the algorithm is designed to reconstruct an image from multiple images of the same scenario but with different shear angles. Changing the shear direction in a microscope is not possible. The microscope stage can be moved though, however if cells are not fixed and get shifted, then matching the corresponding images becomes a challenging task and generates a new problem. For a single DIC image the equation to get the reconstructed image is the following:


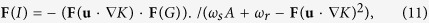


where *A* is the Fourier transform of the 2D discrete Laplace operator and./ is the point-wise division. This algorithm is referred to as ‘Yin’.

### Sparseness-enhanced multiplicative update (SEMU)

The reconstruction task can be formulated as a linear equation system^16^. Let **H** note the transfer matrix of the image formation model, **g** note *G* and **f** note *I* by converting them to column vectors. The transfer matrix **H** has to be matched with the column- or row-order conversion of the variables *G* and *I*. The general objective function can be written as:


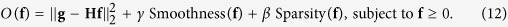


The smoothness term in the objective function forces the reconstruction to have smooth areas, while the sparsity term makes the pixel intensities in the reconstruction to have small values, close to zero.

The described algorithm differs from the original such that the flat-field correction^31^ step is ignored. Bias-correction is a different field and not performing it makes the comparison of the algorithms fair.

One special form of the objective function uses L2 norm and weighted L1 sparsity besides the data term. The matrix **R** stands for the smoothness term which represents the Laplace operator. The matrix **W** which stands for the sparsity term is a diagonal matrix with positive weights on the diagonals and zeros elsewhere. The objective function is written as:


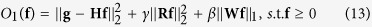


Equation (13) can be expressed as a nonnegative-constrained quadratic program. The algorithm ensures that the non-negativity of the result is preserved during the iterations. The weights in **W** can optionally be updated during the iterations by a log-sum penalty function, which can speed up the convergence. This algorithm is referred to as Sparseness-Enhanced Multiplicative Update (SEMU).

### Second order cone program (SOCP)

The general objective function written in equation (12) can be specialized such that it incorporates TV smoothness term instead of L2 regularization which is used in equation (13). Furthermore, the weighting function from the sparsity term can be removed. The objective function becomes:


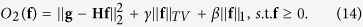


Equation (14) can be solved as a second-order cone program^16^ using a general-purpose convex optimization tool^32^.

### An earlier variational framework

Previously an energy function for DIC image reconstruction was formulated as^25^:


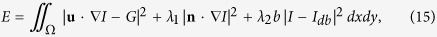


where **n** is the normal vector of the shear direction, *b* is a binary function that indicates whether a pixel belongs to the background or not, *λ*_1_ and *λ*_2_ are weight constants and *I*_*db*_ is the desired background pixel value. The directional derivative perpendicular to the shear removes the bright stripes that appear in the shear direction as artifacts due to noise and the imperfection of the image formation model. Determining variable *b* is generally impossible since objects can appear anywhere in the image. One way is to use the smoothed version of the variance filtered DIC image or its thresholded version for *b*, but it does not guarantee a correct solution. The Euler equation of the energy function is shown in equation (16).


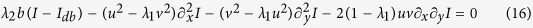


Let ‘Feineigle’ refer to this algorithm, which solves the problem by iteratively updating the reconstruction based on this function.

## Additional Information

**How to cite this article**: Koos, K. *et al*. DIC image reconstruction using an energy minimization framework to visualize optical path length distribution. *Sci. Rep*. **6**, 30420; doi: 10.1038/srep30420 (2016).

## Supplementary Material

Supplementary Information
