# Supplementary material for: DIC image reconstruction using an energy minimization framework to visualize optical path length distribution
Source: Sci Rep. 2016 Jul 25;6:30420. doi: 10.1038/srep30420 (PMC4958949; doi:10.1038/srep30420)
Supplement: Supplementary Information [file srep30420-s1.pdf]

Supplementary Information

Manuscript title: DIC image reconstruction using an energy minimization framework to visualize optical path length distribution

Authors: Krisztian Koos[1], József Molnár[1], Lóránd Kelemen[2], Gábor Tamás[3], Peter Horvath[1,4]

Affiliations:

1: Synthetic and Systems Biology Unit, Hungarian Academy of Sciences, BRC, Szeged, Hungary

2: Institute of Biophysics, Hungarian Academy of Sciences, BRC, Szeged, Hungary

3: MTA-SZTE Research Group for Cortical Microcircuits, Department of Physiology, Anatomy and Neuroscience, University of Szeged, Szeged, Hungary

4: Institute for Molecular Medicine Finland (FIMM), University of Helsinki, Helsinki, Finland

Supplementary Discussion 1: Comparison of DIC simulation models

In this appendix, we show that visually similar results can be obtained by the linear image formation model used in the main paper and the optically accurate model implemented in *microlith*[1]. The table below contains three result images to visually compare the two models and the corresponding parameters we used for the generation. The parameter of the linear model is the σ value, while the parameter for microlith is the Numerical Aperture (NA) value. Other microscope related parameters were unchanged between the simulations. First, we have selected frequent NA values. Then on the synthetic images (without rotation) we have searched for the best correlating sigma value and averaged the results. We observed very high correlation between the two models (the correlation coefficient was usually between 0.97 and 0.99, the least correlation observed was 0.9566). Note, that the linear model has two limitations that this comparison does not cover. First, the simplified model assumes that the examined objects are purely phase-objects and possible amplitude change is not considered. The second is that diffraction is not observable in these images. Diffraction can produce halos around the objects that negatively affect all of the studied algorithms. However, if the linear model is assumed (ie. the sample is phase-object and no diffraction occurs) the presented comparison of the models is valid. Furthermore, since the linear model is assumed all over the tests presented in the main paper, it allows the algorithms to be compared under equal conditions.

	NA = 0.95	NA = 1.25	NA = 1.4
microlith	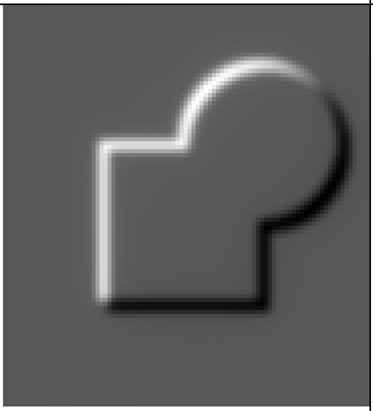	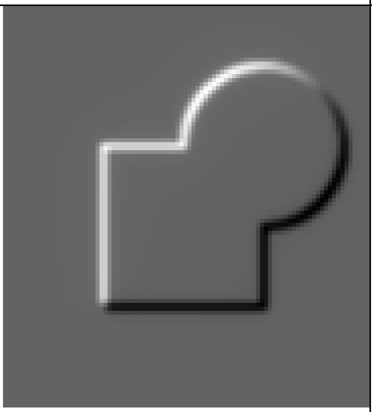	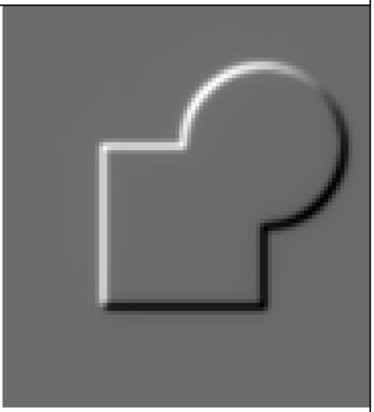
Gaussian derived PSF	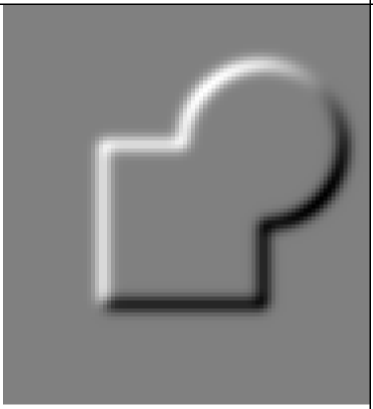	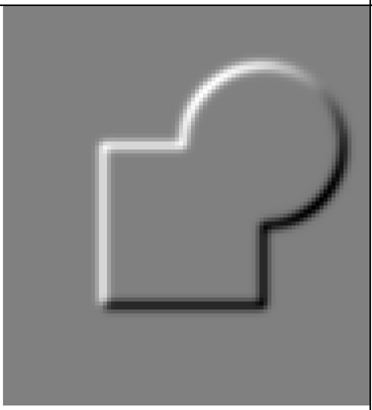	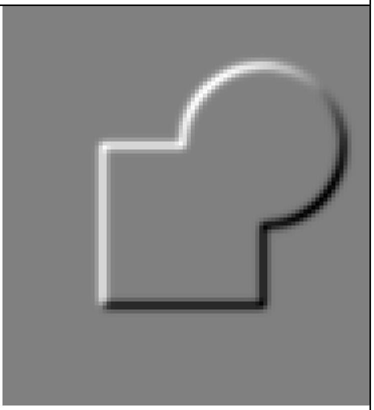
	$\sigma = 2.3$	$\sigma = 1.6$	$\sigma = 1.4$
	corr = 0.9639	corr = 0.9838	corr = 0.9921

References:

1 Mehta, S. B. & Oldenbourg, R. Image simulation for biological microscopy: microlith. *Biomedical optics express* **5**, 1822-1838 (2014).

Supplementary Discussion 2:

Derivation of the Euler-Lagrange equations

Formalizing the problem

In many cases, the simple interpretation of the DIC images as directional derivatives doesn't provide sufficient result. Here we assume that this basic model suffers from oversimplification, because the point spread function isn't correctly modeled or not incorporated at all. Directional derivative of the intensity function I defined with non-unit directional vector $\mathbf{v}$ such that $\nabla I \cdot \mathbf{v} = I_x v + I_y w,\ \nabla I = \begin{bmatrix} I_x & I_y \end{bmatrix}^T, \mathbf{v} = \begin{bmatrix} v & w \end{bmatrix}^T$. This is approximately (using Lagrange mean value theorem) $I\left(x+\frac{v}{2}, y+\frac{w}{2}\right) - I\left(x-\frac{v}{2}, y-\frac{w}{2}\right)$, i.e. a directional difference. Difference in one dimension can also be defined with the integral $\int\limits_{\xi=x-\delta}^{\xi=x+\delta} I'(\xi)d\xi = I(x+\delta) - I(x-\delta)$ where the left hand side can be written: $\int\limits_{x-\delta}^{x+\delta} I'dx = \int\limits_{x-\delta}^{x+\delta} 1I'dx$.

This last form suggest the trivial generalization using arbitrary kernel $K(\xi)$ such that $\int\limits_{x-\delta}^{x+\delta} 1I'(\xi)d\xi \to \int\limits_{x-\delta}^{x+\delta} K(\xi - x)I'(\xi)d\xi$, where the (finite) domain of K is $[-\delta, \delta]$.

Based on the consideration above, we propose to model the point spread phenomenon with integral transform, i.e. we assume that the DIC image (G_0) is actually the derivative of the transformed function with some appropriately chosen symmetric, finite and constant size (independent of x and y) K kernel:

$$\begin{aligned}
\hat{I}(x,y) &= \iint\limits_{\{-\delta,\delta\},\{-\delta,\delta\}} K(\xi,\eta) I(x+\xi, y+\eta) d\xi d\eta \\
&\left(= \iint\limits_{\{x-\delta,x+\delta\},\{y-\delta,y+\delta\}} K(\xi - x, \eta - y) I(\xi,\eta) d\xi d\eta \right) \\
&\Rightarrow \\
\hat{I}_x(x,y) &= \iint\limits_{\xi,\eta} I_x(x+\xi, y+\eta) K(\xi,\eta) d\xi d\eta \\
\hat{I}_y(x,y) &= \iint\limits_{\xi,\eta} I_y(x+\xi, y+\eta) K(\xi,\eta) d\xi d\eta
\end{aligned} \tag{I.1}$$

where K is often chosen the trimmed version of the Gaussian kernel (the density function of the normal distribution) with standard deviation σ as

$K(\xi,\eta)=\frac{1}{2\pi\sigma^2}e^{-\frac{\xi^2+\eta^2}{2\sigma^2}}$.[1]

A functional to be minimized is then written such:

$$
\begin{aligned}
E(\nabla I) &\doteq \frac{1}{2}\int_{\Omega}\left[\mathbf{u}_0\cdot\iint_{\xi,\eta}\nabla I(x+\xi,y+\eta)K(\xi,\eta)d\xi d\eta-(G_0-c)\right]^2 dA \\
&= \frac{1}{2}\iint_{x,y}\left[\iint_{\xi,\eta}(u_0 I_x+v_0 I_y)Kd\xi d\eta-(G_0-c)\right]^2 dxdy
\end{aligned}
\tag{I.2}
$$

where I is the sought intensity function, $\mathbf{u}_0=[u_0,v_0]^T$, $|\mathbf{u}_0|=1$ is the predefined direction, G_0 is the available DIC image, and c is a constant.

To calculate the Euler-Lagrange equation, first the variation of the functional must be determined, then its directional derivative (by finite directional derivative analogy). For comparison 1st we see how can it be done for the basic (without kernel) variant:

$$
\begin{aligned}
&\left.\frac{\partial}{\partial\varepsilon}\right|_{\varepsilon=0}\frac{1}{2}\iint_{x,y}\left\{\left[u_0(I_x+\varepsilon h_x)+v_0(I_y+\varepsilon h_y)\right]-(G_0-c)\right\}^2 dxdy= \\
&\iint_{x,y}\left[(u_0 I_x+v_0 I_y)-(G_0-c)\right](u_0 h_x+v_0 h_y)dxdy= \\
&\iint_{x,y}h_x u_0\left[(u_0 I_x+v_0 I_y)-(G_0-c)\right]dxdy+\iint_{x,y}h_y v_0\left[(u_0 I_x+v_0 I_y)-(G_0-c)\right]dxdy \\
&\Rightarrow \\
&-\iint_{x,y}h\frac{\partial}{\partial x}\left[u_0(u_0 I_x+v_0 I_y)-u_0(G_0-c)\right]dxdy-\iint_{x,y}h\frac{\partial}{\partial y}\left[v_0(u_0 I_x+v_0 I_y)-v_0(G_0-c)\right]dxdy= \\
&-\iint_{x,y}h\left\{\frac{\partial}{\partial x}\left[u_0(u_0 I_x+v_0 I_y)-u_0(G_0-c)+\frac{\partial}{\partial y}\left[v_0(u_0 I_x+v_0 I_y)-v_0(G_0-c)\right]\right]\right\}dxdy
\end{aligned}
\tag{I.3}
$$

The associated Euler-Lagrange equation is under the brace with the constant c eliminated by derivation:

$$
\begin{gathered}
\frac{\partial}{\partial x}\left[u_0(u_0 I_x+v_0 I_y)-u_0 G_0+\frac{\partial}{\partial y}\left[v_0(u_0 I_x+v_0 I_y)-v_0 G_0\right]\right]=0 \\
\Leftrightarrow \\
\mathbf{u}_0\cdot\nabla\nabla I\cdot\mathbf{u}_0-\mathbf{u}_0\cdot\nabla G_0=0
\end{gathered}
\tag{I.4}
$$

Next we turn our attention to the Lagrangian partly given by local integrals:

$L\doteq\left[-(G_0(x,y)-c)+\iint_{\xi,\eta}(u_0 I_x(x+\xi,y+\eta)+v_0 I_y(x+\xi,y+\eta))K(\xi,\eta)d\xi d\eta\right]^2\doteq S^2$, from which:

[1] The window size should be about 4-6 times of σ (i.e. 3x3 for σ≤1 5x5 for 1≤σ≤1.5, 7x7 for 1.5≤σ≤2 …)

$$
\begin{aligned}
&0=\frac{\partial}{\partial\varepsilon}\bigg|_{\varepsilon=0}\frac{1}{2}\iint_{x,y}\left[\iint_{\xi,\eta}\left[u_0\left(I_x+\varepsilon h_x\right)+v_0\left(I_y+\varepsilon h_y\right)\right]Kd\xi d\eta-\left(G_0-c\right)\right]^2dxdy=\\
&\iint_{x,y}\left[\iint_{\xi,\eta}K\left(u_0h_x+v_0h_y\right)d\xi d\eta\right]\left[\iint_{\xi,\eta}\left(u_0I_x+v_0I_y\right)Kd\xi d\eta-\left(G_0-c\right)\right]dxdy=\\
&\iint_{x,y}\left[u_0\iint_{\xi,\eta}Kh_xd\xi d\eta+v_0\iint_{\xi,\eta}Kh_yd\xi d\eta\right]Sdxdy=\\
&\iint_{x,y}\left[u_0\iint_{\xi,\eta}K\sum_{m,n=0}^{\infty}\frac{1}{m!n!}\frac{\partial^{m+n}h_x}{\partial x^m\partial y^n}\xi^m\eta^n\;d\xi d\eta+v_0\iint_{\xi,\eta}K\sum_{m,n=0}^{\infty}\frac{1}{m!n!}\frac{\partial^{m+n}h_y}{\partial x^m\partial y^n}\xi^m\eta^n\;d\xi d\eta\right]Sdxdy=\\
&\iint_{x,y}\left[u_0\sum_{m,n=0}^{\infty}\frac{1}{m!n!}\frac{\partial^{m+n+1}h}{\partial x^{m+1}\partial y^n}\iint_{\xi,\eta}K\xi^m\eta^nd\xi d\eta+v_0\sum_{m,n=0}^{\infty}\frac{1}{m!n!}\frac{\partial^{m+n+1}h}{\partial x^m\partial y^{n+1}}\iint_{\xi,\eta}K\xi^m\eta^nd\xi d\eta\right]Sdxdy= \qquad \text{(I.5)}\\
&\iint_{x,y}\left[\sum_{m,n=0}^{\infty}\frac{1}{m!n!}\frac{\partial^{m+n+1}h}{\partial x^{m+1}\partial y^n}\iint_{\xi,\eta}K\xi^m\eta^nd\xi d\eta\right]u_0Sdxdy+\\
&\iint_{x,y}\left[\sum_{m,n=0}^{\infty}\frac{1}{m!n!}\frac{\partial^{m+n+1}h}{\partial x^m\partial y^{n+1}}\iint_{\xi,\eta}K\xi^m\eta^nd\xi d\eta\right]v_0Sdxdy\qquad\overset{\text{using partial integration steps}}{\Rightarrow}\\
&0=\iint_{x,y}h\left[u_0\sum_{m,n=0}^{\infty}\frac{(-1)^{m+n+1}}{m!n!}\left(\iint_{\xi,\eta}K\xi^m\eta^nd\xi d\eta\right)\frac{\partial^{m+n+1}S}{\partial x^{m+1}\partial y^n}\right]dxdy+\\
&\quad\iint_{x,y}h\left[v_0\sum_{m,n=0}^{\infty}\frac{(-1)^{m+n+1}}{m!n!}\left(\iint_{\xi,\eta}K\xi^m\eta^nd\xi d\eta\right)\frac{\partial^{m+n+1}S}{\partial x^m\partial y^{n+1}}\right]dxdy
\end{aligned}
$$

The Euler-Lagrange equation

From (I.5), the Euler-Lagrange equations are:

$$
\begin{aligned}
&\sum_{m,n=0}^{\infty}\frac{(-1)^{m+n+1}}{m!n!}\left(\iint_{\xi,\eta}K(\xi,\eta)\xi^m\eta^nd\xi d\eta\right)\left(u_0\frac{\partial^{m+n+1}S}{\partial x^{m+1}\partial y^n}+v_0\frac{\partial^{m+n+1}S}{\partial x^m\partial y^{n+1}}\right)=0\\
&S=-\left(G_0(x,y)-c\right)+\iint_{\xi,\eta}\left(u_0I_x(x+\xi,y+\eta)+v_0I_y(x+\xi,y+\eta)\right)K(\xi,\eta)d\xi d\eta
\end{aligned}
\qquad \text{(I.6)}
$$

The first ('piecewise constant') approximation of the Euler Lagrange equation and the gradient descent (with α scaling factor):

$$
\begin{array}{l}
m = n = 0 \\
\Rightarrow \\
\left(\iint\limits_{\xi,\eta} K(\xi,\eta) d\xi d\eta \right) \left(u_0 \frac{\partial S}{\partial x} + v_0 \frac{\partial S}{\partial y} \right) = 0 \\
\Leftrightarrow \\
-u_0 \left(\iint\limits_{\xi,\eta} \left(u_0 I_{xx}(x+\xi, y+\eta) + v_0 I_{xy}(x+\xi, y+\eta) \right) K(\xi,\eta) d\xi d\eta - G_{0x}(x,y) \right) \\
-v_0 \left(\iint\limits_{\xi,\eta} \left(u_0 I_{xy}(x+\xi, y+\eta) + v_0 I_{yy}(x+\xi, y+\eta) \right) K(\xi,\eta) d\xi d\eta - G_{0y}(x,y) \right) = 0 \\
\Leftrightarrow \\
-\iint\limits_{\xi,\eta} \mathbf{u}_0 \cdot \nabla\nabla I \cdot \mathbf{u}_0 K d\xi d\eta + \nabla G_0 \cdot \mathbf{u}_0 = 0 \quad \Rightarrow \quad \frac{\partial I}{\partial \tau} = \alpha \left(-\nabla G_0 \cdot \mathbf{u}_0 + \iint\limits_{\xi,\eta} K \mathbf{u}_0 \cdot \nabla\nabla I \cdot \mathbf{u}_0 d\xi d\eta \right)
\end{array}
\tag{I.7}
$$

is very much like the basic (non-kernel) equations. The 'bilinear' equations:

$$
\begin{aligned}
0 = &-\left(\iint\limits_{\xi,\eta} K(\xi,\eta) d\xi d\eta \right) \left(u_0 \frac{\partial S}{\partial x} + v_0 \frac{\partial S}{\partial y} \right) \\
&+\left(\iint\limits_{\xi,\eta} K(\xi,\eta) \xi d\xi d\eta \right) \left(u_0 \frac{\partial^2 S}{\partial x^2} + v_0 \frac{\partial^2 S}{\partial x \partial y} \right) + \left(\iint\limits_{\xi,\eta} K(\xi,\eta) \eta d\xi d\eta \right) \left(u_0 \frac{\partial^2 S}{\partial x \partial y} + v_0 \frac{\partial^2 S}{\partial y^2} \right) \\
&-\left(\iint\limits_{\xi,\eta} K(\xi,\eta) \xi\eta d\xi d\eta \right) \left(u_0 \frac{\partial^3 S}{\partial x^2 \partial y} + v_0 \frac{\partial^3 S}{\partial x \partial y^2} \right)
\end{aligned}
\tag{I.8}
$$

or rearranging for u_0 and v_0:

$$
\begin{aligned}
0 = u_0 &\left[-\frac{\partial S}{\partial x} \iint\limits_{\xi,\eta} K(\xi,\eta) d\xi d\eta + \frac{\partial^2 S}{\partial x^2} \iint\limits_{\xi,\eta} K(\xi,\eta) \xi d\xi d\eta + \frac{\partial^2 S}{\partial x \partial y} \iint\limits_{\xi,\eta} K(\xi,\eta) \eta d\xi d\eta - \frac{\partial^3 S}{\partial x^2 \partial y} \iint\limits_{\xi,\eta} K(\xi,\eta) \xi\eta d\xi d\eta \right] \\
+ v_0 &\left[-\frac{\partial S}{\partial y} \iint\limits_{\xi,\eta} K(\xi,\eta) d\xi d\eta + \frac{\partial^2 S}{\partial x \partial y} \iint\limits_{\xi,\eta} K(\xi,\eta) \xi d\xi d\eta + \frac{\partial^2 S}{\partial y^2} \iint\limits_{\xi,\eta} K(\xi,\eta) \eta d\xi d\eta - \frac{\partial^3 S}{\partial x \partial y^2} \iint\limits_{\xi,\eta} K(\xi,\eta) \xi\eta d\xi d\eta \right]
\end{aligned}
\tag{I.9}
$$

where the derivatives of S, $m,n \in \{1,2\}$:

$$
\frac{\partial^{m+n} S}{\partial x^m \partial y^n} = -\frac{\partial^{m+n} G_0}{\partial x^m \partial y^n} + \iint\limits_{\xi,\eta} \left(u_0 \frac{\partial^{m+n+1} I}{\partial x^{m+1} \partial y^n} + v_0 \frac{\partial^{m+n+1} I}{\partial x^m \partial y^{n+1}} \right) K(\xi,\eta) d\xi d\eta
\tag{I.10}
$$

The plausible assumption here is that higher window (higher σ) requires higher-order approximation, e.g. $\sigma \le 1.5$ (3x3 to 5x5 window size) (I.7) may be used and for higher deviation (5x5 to 7x7 window size) (I.9-10) may be used.

Alternative equation

The equivalent expression is given in (I.1), second line in brackets:

$$
\begin{aligned}
\hat{I}\left(x,y\right) &= \iint\limits_{\left\{x-\delta,x+\delta\right\},\left\{y-\delta,y+\delta\right\}} K\left(\xi-x,\eta-y\right)I\left(\xi,\eta\right)d\xi d\eta \qquad \rightarrow \\
\hat{I}\left(x+dx,y\right) &= \iint\limits_{\left\{x+dx-\delta,x+dx+\delta\right\},\left\{y-\delta,y+\delta\right\}} K\left(\xi-x-dx,\eta-y\right)I\left(\xi,\eta\right)d\xi d\eta \\
&\approx \iint\limits_{\left\{x+dx-\delta,x+dx+\delta\right\},\left\{y-\delta,y+\delta\right\}} \left(K-\frac{\partial K}{\partial x}dx\right)I\left(\xi,\eta\right)d\xi d\eta \\
&= \iint\limits_{\left\{x-\delta,x+\delta\right\},\left\{y-\delta,y+\delta\right\}} \left(K-\frac{\partial K}{\partial x}dx\right)I\left(\xi,\eta\right)d\xi d\eta \\
&+ \iint\limits_{\left\{x+\delta,x+\delta+dx\right\},\left\{y-\delta,y+\delta\right\}} \left(K-\frac{\partial K}{\partial x}dx\right)I\left(\xi,\eta\right)d\xi d\eta \\
&- \iint\limits_{\left\{x-\delta,x-\delta+dx\right\},\left\{y-\delta,y+\delta\right\}} \left(K-\frac{\partial K}{\partial x}dx\right)I\left(\xi,\eta\right)d\xi d\eta \\
&\approx \iint\limits_{\left\{x-\delta,x+\delta\right\},\left\{y-\delta,y+\delta\right\}} K\Big|_{\xi-x,\eta-y} I\left(\xi,\eta\right)d\xi d\eta \\
&- \iint\limits_{\left\{x-\delta,x+\delta\right\},\left\{y-\delta,y+\delta\right\}} \frac{\partial K}{\partial x}\bigg|_{\xi-x,\eta-y} dxI\left(\xi,\eta\right)d\xi d\eta \\
&+ \int\limits_{\left\{y-\delta,y+\delta\right\}} dx\left(K-\frac{\partial K}{\partial x}dx\right)\bigg|_{\xi+x,\eta-y} I\left(\xi,\eta\right)d\eta \\
&- \int\limits_{\left\{y-\delta,y+\delta\right\}} dx\left(K-\frac{\partial K}{\partial x}dx\right)\bigg|_{\xi-x,\eta-y} I\left(\xi,\eta\right)d\eta \\
&\Rightarrow \\
\frac{\hat{I}\left(x+dx,y\right)-\hat{I}\left(x,y\right)}{dx} &\approx \iint\limits_{\left\{x-\delta,x+\delta\right\},\left\{y-\delta,y+\delta\right\}} \frac{\partial K}{\partial x}\bigg|_{\xi-x,\eta-y} I\left(\xi,\eta\right)d\xi d\eta \\
&+ \int\limits_{\left\{y-\delta,y+\delta\right\}} \left(K-\frac{\partial K}{\partial x}dx\right)\bigg|_{\xi+x,\eta-y} I\left(\xi,\eta\right)d\eta \\
&- \int\limits_{\left\{y-\delta,y+\delta\right\}} \left(K-\frac{\partial K}{\partial x}dx\right)\bigg|_{\xi-x,\eta-y} I\left(\xi,\eta\right)d\eta
\end{aligned} \tag{I.11}
$$

Taking the limit:

$$
\begin{aligned}
\frac{\hat{I}\left(x+dx,y\right)-\hat{I}\left(x,y\right)}{dx} &= \frac{\partial\hat{I}\left(x,y\right)}{dx}\bigg|_{x,y} \\
&= \iint\limits_{\left\{x-\delta,x+\delta\right\},\left\{y-\delta,y+\delta\right\}} \frac{\partial K}{\partial x}\left(\xi-x,\eta-y\right)I\left(\xi,\eta\right)d\xi d\eta \\
&+ \int\limits_{\left\{y-\delta,y+\delta\right\}} K\left(\xi+x,\eta-y\right)I\left(\xi,\eta\right)d\eta \\
&- \int\limits_{\left\{y-\delta,y+\delta\right\}} K\left(\xi-x,\eta-y\right)I\left(\xi,\eta\right)d\eta
\end{aligned} \tag{I.12}
$$

The last two terms are eliminated if they are zero at the window boundary (we also suppose that their derivatives are zero as well).[2] Gauss kernel is the good approximation for such a kernel. In this case:

[2] This condition is not necessary, but we require it for the higher derivatives too.

$$
\begin{aligned}
\hat{I}(x,y) &= \iint_{\{-\delta,\delta\},\{-\delta,\delta\}} K(\xi,\eta) I(x+\xi, y+\eta) d\xi d\eta \\
&= \iint_{\{x-\delta,x+\delta\},\{y-\delta,y+\delta\}} K(\xi-x,\eta-y) I(\xi,\eta) d\xi d\eta \\
&\Rightarrow \\
\frac{\partial \hat{I}}{\partial x}(x,y) &= \iint_{\{-\delta,\delta\},\{-\delta,\delta\}} \frac{\partial I}{\partial x}(\xi+x,\eta+y) K(\xi,\eta) d\xi d\eta \\
&= \iint_{\{x-\delta,x+\delta\},\{y-\delta,y+\delta\}} \frac{\partial K}{\partial x}(\xi-x,\eta-y) I(\xi,\eta) d\xi d\eta
\end{aligned}
\tag{I.13}
$$

Or for higher-order derivatives, hence (I.10):

$$
\begin{aligned}
&\left(\frac{\partial^{m+n} \hat{I}}{\partial x^m \partial y^n}(x,y) = \right) \\
&\iint_{\substack{\{-\delta,\delta\} \\ \{-\delta,\delta\}}} \frac{\partial^{m+n} I}{\partial x^m \partial y^n}(\xi+x,\eta+y) K(\xi,\eta) d\xi d\eta = \iint_{\substack{\{x-\delta,x+\delta\} \\ \{y-\delta,y+\delta\}}} \frac{\partial^{m+n} K}{\partial x^m \partial y^n}(\xi-x,\eta-y) I(\xi,\eta) d\xi d\eta \\
&\Rightarrow \\
&\frac{\partial^{m+n} S}{\partial x^m \partial y^n} = -\frac{\partial^{m+n} G_0}{\partial x^m \partial y^n} + \iint_{\substack{\{x-\delta,x+\delta\} \\ \{y-\delta,y+\delta\}}} \left(u_0 \frac{\partial^{m+n+1} K}{\partial x^{m+1} \partial y^n} + v_0 \frac{\partial^{m+n+1} K}{\partial x^m \partial y^{n+1}} \right)\Bigg|_{\xi-x,\eta-y} I\Big|_{(\xi,\eta)} d\xi d\eta
\end{aligned}
\tag{I.14}
$$
